# Effects of maternal supplementation with fatty acid-balanced oil on the performance of sows and intestinal barrier function of offspring

**DOI:** 10.1016/j.aninu.2026.04.009

**Published:** 2026-09-09

**Authors:** Huiling Zhu, Yuhui Tang, Kun Qin, Mengke Yan, Yu Cao, Jihua Wang, Xiao Xu, Shaokui Chen, Kan Xiao, Yulan Liu

**Affiliations:** a Hubei Key Laboratory of Animal Nutrition and Feed Science, Wuhan Polytechnic University, Wuhan 430023, Hubei, China; b Calid Biotech (Wuhan) Co., Ltd., Wuhan 430074, Hubei, China

**Keywords:** Fatty acid-balanced oil, Growth performance, Intestinal barrier function, Sow, Offspring

## Abstract

Dietary lipid supplementation improves sow reproductive performance, survival, and growth of piglets. This study was conducted to investigate the effects of maternal fatty acid-balanced oil (FABO) supplementation during late gestation and lactation on the reproductive performance of sows, and the growth performance and intestinal barrier function of their offspring. Twenty-four multiparous sows (Landrace × Large White) with similar parity (2.29 ± 0.25), body weight (BW; 223.60 ± 11.32 kg), and backfat (BF) thickness (17.10 ± 2.03 mm) were divided into 2 treatment groups (*n* = 12). The sows were fed with a basal diet supplemented with either 2% soybean oil (2% SO) or 2% FABO from d 90 of gestation to the end of lactation (d 21 postpartum). At d 21 postpartum, 24 piglets (one per litter, 5.32 ± 0.07 kg BW, six per group) were assigned to a 2 × 2 factorial arrangement of treatments (2% SO vs. 2% FABO) and lipopolysaccharide (LPS) challenge (saline vs. LPS). Within each maternal diet group, half of the piglets were injected intraperitoneally with either 100 μg/kg BW LPS or saline. At 4 h post-injection, all piglets were slaughtered and intestinal samples were collected. Maternal 2% FABO supplementation increased the piglet weight at birth and litter weight at weaning (*P* < 0.05), and tended to enhance weaning survivability (*P* = 0.076). Moreover, piglets born to sows supplemented with 2% FABO exhibited higher litter weight gain and average daily gain compared to those from sows receiving 2% SO diet (*P* < 0.05). The 2% FABO increased colostral protein, immunoglobulin (Ig) G, IgM, and IgA concentrations (*P* < 0.05). Piglets suckling 2% FABO-supplemented sows exhibited greater villus height and maltase and sucrase activities in the jejunum (*P* < 0.05). Maternal dietary 2% FABO supplementation fortified intestinal barrier function, as evidenced by elevated goblet cell number and intestinal tight junction protein claudin-1 and ZO-1 expression, as well as mRNA abundance of *pBD-3* and secretory immunoglobulin A (sIgA) concentration in the jejunum of piglets (*P* < 0.05). Piglets suckling 2% FABO-supplemented sows had lower mRNA abundance of *HSP-70* and higher *IL-*10 mRNA abundance in the jejunum (*P* < 0.05). Sow dietary 2% FABO supplementation elevated the IL-22/STAT3 pathway key factors *JAK1*, *TYK2,* and *STAT3* mRNA abundance, and IL-22, p-STAT3 protein expression, and p-STAT3:t-STAT3 ratio in the jejunum of piglets (*P* < 0.05). In conclusion, maternal supplementation with 2% FABO increased IgG, IgM, and IgA concentrations in colostrum, and improved growth performance of offspring. Moreover, maternal dietary 2% FABO supplementation fortified intestinal barrier function of offspring, likely through modulation of the IL-22/STAT3 signaling pathway.

## 1 Introduction

Sow reproductive outcomes and neonatal piglet growth performance are decisive factors determining the sustainable development of the swine industry (Chen et al., 2019). Maternal nutrition status significantly influences the development of fetal organ systems (Funston and Vonnahme, 2010). During late gestation and lactation, the nutritional requirements of sows increase substantially due to the rapid growth of fetuses and the demands of milk production (Kim et al., 2013; Tokach et al., 2019). However, sows often cannot obtain sufficient energy from feed due to low feed intake, and must mobilize body reserves to meet their energy requirements (Holen et al., 2023). Excessive mobilization of body tissue compromises the subsequent fertility of sows and reduces offspring survival rate (Costermans et al., 2020; Tritton et al., 1996). Strategically, dietary lipid supplementation during late gestation and lactation has been demonstrated to enhance sow reproductive performance, and improve piglet survival and growth (Gatlin et al., 2002; Laws et al., 2018; Wang et al., 2022).

Lipids are vital in the nutrition of sows by serving as a primary energy source and supplying essential fatty acids. These fatty acids perform diverse biological functions, including the regulation of tissue structure and functions, as well as antibacterial and anti-inflammatory activities (Fan et al., 2022; Liu, 2016). Specifically, dietary fatty acids benefited both sow reproductive performance and piglet performance (birth weight and suckling growth) (Rosero et al., 2015; Zentak et al., 2011). Different classes of fatty acids demonstrate distinct benefits. For instance, butyric acid has been shown to enhance lactation performance in sows and accelerate pre-weaning growth in their offspring (Qi et al., 2023). Sow diets supplemented with medium-chain fatty acid monoglyceride or medium-chain fatty acid triglycerides (MCTs) during gestation and lactation enhanced the intestinal barrier function and increased the survival rates of piglets (Azain, 1993; Gatlin et al., 2002; Zhao et al., 2023). In addition, long-chain polyunsaturated fatty acids (PUFAs) are indispensable to embryogenesis and fetal programming (Tanghe et al., 2015). Notably, dietary n-3 PUFA supplementation modulated the weaning-to-estrus interval positively in sows, while enhancing maternal immune status and stimulating piglet growth rate (Baidoo et al., 2003; Desaldeleer et al., 2014; Tanghe et al., 2015).

Fatty acid composition fundamentally dictates the nutritional properties of lipids. Imbalanced fatty acids intake in modern diets has been identified as a primary causative factor for cardiovascular diseases (Uriho et al., 2019). Moreover, in swine production, an imbalanced n-6:n-3 PUFA ratio in commercial sow diets is correlated with decreased piglet survival at birth and subsequent growth (Rooke et al., 2001). Reducing the n-6:n-3 PUFAs ratio in sow diets improved offspring weaning weight, survival and weight gain (Nguyen et al., 2020). Furthermore, increasing the unsaturated fatty acid (UFA) to saturated fatty acid (SFA) ratio improved the feed efficiency of gilts (Becker and Greiner, 2023). Accordingly, the World Health Organization recommends limiting the intake of SFAs and trans fats, concurrently promoting the intake of monounsaturated fatty acids (MUFAs), PUFAs and considerable amounts of n-3 PUFA, to prevent fatty acid imbalance-induced pathologies (Uriho et al., 2019). Key aspects of fatty acid balance include the ratio of UFAs to SFAs, the n-3:n-6 PUFAs ratio, and the proportion of short- and medium-chains to long chain fatty acids (Uriho et al., 2019; Zhang et al., 2024).

In fact, no single type of oil can fulfill the requirement of a balanced fatty acid profile (Bian et al., 2022). Mixing various oils in a specific ratio is one of the most efficient approaches to producing blended oils with an optimal fatty acid pattern (Bian et al., 2022; Rabail et al., 2024). Hence, fatty acid-balanced oil (FABO) was developed in the present study by blending tributyrin, coconut oil, palm oil, flaxseed oil, and soybean oil (SO) in appropriate proportions. The objective of this study was to evaluate the effects of maternal FABO supplementation during late gestation and lactation on sow reproductive performance, and the growth performance and intestinal barrier function of offspring.

## 2 Material and method

### 2.1 Animal ethics statement

The study was approved by the Animal Care and Use Committee of Wuhan Polytechnic University (approval No. WPU202304004; approval date, April 4, 2023).

### 2.2 Animal and experimental design

Twenty-four multiparous sows (d 90 of gestation, Landrace × Large White) with similar parity (2.29 ± 0.25), body weight (BW; 223.60 ± 11.32 kg), and backfat (BF) thickness (17.10 ± 2.03 mm) were randomly divided into 2 dietary treatments with 12 replicates (1 sow per replicate pen). The dietary treatments were 1) basal diet supplemented with 2% SO, and 2) basal diet supplemented with 2% FABO. The NRC (2012) recommends that the background fat level in sow diets, without additional supplementation is approximately 2%. Therefore, both FABO and SO were supplemented at 2% in this study. Previous studies have recommended balanced fatty acid ratios as follows: a ratio of (2.25–3.50):1.00 for UFAs to SFAs and a ratio of (5–10):1 for n-3 to n-6 PUFAs (Eastwood et al., 2014; Gatlin et al., 2002; Tartrakoon et al., 2016; Villagómez-Estrada et al., 2024; Yao et al., 2012). Currently, the ratio of short- and medium-chain to long-chain fatty acids in FABO has not been reported. Previous studies indicated that the ratio of short- and medium-chain to long-chain fatty acids in porcine colostrum and milk was 0.00 to 0.07 (Azain, 1993; Lauridsen and Danieslen, 2004; Zhang et al., 2024). Based on these studies, FABO was prepared by combining tributyrin (3.33%), coconut oil (3.33%), palm oil (20.5%), SO (66.17%), and flaxseed oil (6.67%), then homogenizing the mixture for approximately 60 min at a constant 40 °C, as described by Grover et al. (2021). All the oils used in this study were provided by Calid Biotech (Wuhan) Co., Ltd. (Wuhan, Hubei, China). The fatty acid composition of SO and FABO is shown in Table 1.

**Table 1:** Fatty acid composition and content (mg/g) of the SO and the FABO^1^.

Items	Content	
	SO	FABO
C4:0	0.00	42.64
C6:0	0.00	1.98
C8:0	0.06	0.08
C10:0	0.11	1.90
12:0	0.08	13.88
14:0	0.73	7.37
C15:0	0.15	0.26
C16:0	101.35	152.06
C16:1	0.83	0.89
C17:0	0.94	0.88
C18:0	41.88	40.61
C18:1n-9	208.48	220.24
C18:2n-6	512.10	358.39
C18:3n-3	66.40	70.67
C20:0	3.73	3.16
C20:1	1.90	1.53
C21:0	0.46	0.14
C22:0	4.03	2.55
C22:1n-9	0.17	0.07
C23:0	0.58	0.41
C24:0	1.41	1.08
C24:1	0.00	0.11
SCFA	0.00	42.64
MCFA	0.25	17.83
LCFA	945.13	860.42
SFA	155.51	226.35
UFA	789.87	651.9
n-6 PUFA	512.10	358.39
n-3 PUFA	66.40	70.67
(SCFA + MCFA):LCFA	0.00	0.07
UFA:SFA	5.08	2.88
n-6 PUFA:n-3 PUFA ratio	7.71	5.07

SO  =  soybean oil; FABO  =  fatty acid-balanced oil; SCFA  =  short chain fatty acid; MCFA  =  medium chain fatty acid; LCFA  =  long chain fatty acid; SFA  =  saturated fatty acid; UFA  =  unsaturated fatty acid; PUFA  =  polyunsaturated fatty acid.

1 The dietary fatty acid composition from C4:0 to C24:1(n-9) were analyzed in duplicate.

Corn-soybean meal-basal diets were formulated to meet the NRC (2012) nutrient recommendation for sows. The composition and nutrient levels of diets are shown in Table 2. The nutrient levels of the diet were analyzed following standard protocols. Dry matter (DM) was determined using an electric blast drying oven (DHG-9070A, Shanghai Yiheng Instrument Co., Ltd., Shanghai, China) according to GB/T 6435-2014 (China National Standard, 2014). Crude protein was analyzed via the Kjeldahl method GB/T 6432-2018 (China National Standard, 2018a) using Kjeldahl nitrogen analyzer (K1270, Hanon Future Technology Group Co., Ltd., Jinan, Shandong, China) and crude ash was determined using a furnace (SK2-7-17TPC4, Henan Brother Instrument Co., Ltd., Zhengzhou, Henan, China) according to GB/T 6438-2007 (China National Standard, 2007). Crude fiber was analyzed using fiber measuring instrument (YJ-6434, Yiyan Zhizao Drug Testing Instrument Co., Ltd., Shanghai, China) by the method GB/T 6434-2022 (China National Standard, 2022). Calcium content was analyzed according to GB/T 6436-2018 (China National Standard, 2018b). Total phosphorus was analyzed according to GB/T 6437-2018 (China National Standard, 2018c)using a UV-Visible spectrophotometer (Model UV-2600, Shimadzu Corp., Kyoto, Japan). According to followed the DM, digestible energy, total lysine, and available phosphorus were calculated according to the Feed Composition and Nutritive Values in China (China Feed Database, 2020). The total organic matter content was calculated as follows:

Organicmatter(%)=(DM–Crudeash)×100.

**Table 2:** Composition and nutrient levels of the experimental diets (%, as-fed basis).

Items	Diets	
	2% SO^1^	2% FABO^1^
**Ingredients**		
Corn	52.20	52.20
Wheat bran (17%)	9.00	9.00
Wheat	6.50	6.50
Soybean meal (43%)	16.50	16.50
Expanded soybean (35%)	5.00	5.00
Steam fish meal (67%)	2.00	2.00
Intestinal mucosa protein (50%)	1.25	1.25
Soybean oil	2.00	0.00
FABO	0.00	2.00
Sugar	1.00	1.00
Broken yeast powder	0.25	0.25
Limestone	1.60	1.60
Dicalcium phosphate	1.40	1.40
Salt	0.30	0.30
Premix^2^	1.00	1.00
Total	100.00	100.00
**Nutrients**^3^		
Digestible energy, MJ/kg	13.83	13.80
Crude protein	17.00	17.00
Crude fiber	7.93	8.00
Calcium	0.50	0.50
Total phosphorus	0.85	0.85
Available phosphorus	0.52	0.52
Total lysine	1.10	1.10
Organic matter	82.63	82.56

SO  =  soybean oil; FABO  =  fatty acid-balanced oil.

1 SO, diet supplemented with 2% soybean oil group; FABO, diet supplemented with 2% fatty acid-balanced oil group.

2 Premix provided the following per kg of diet: vitamin A, 9000 IU; vitamin E, 120 IU; vitamin D_3_, 2500 IU; vitamin B_1_, 3 mg; vitamin B_2_, 8 mg; vitamin B_12_, 0.03 mg; nicotinic acid, 60 mg; pantothenic acid, 25 mg; folic acid, 2.5 mg; biotin, 0.3 mg; choline chloride, 600 mg, Cu (CuSO_4_·5H_2_O), 30 mg; Fe (FeSO_4_·H_2_O), 80 mg; Mn (MnSO_4_·5H_2_O), 45 mg; Zn (ZnSO_4_·7H_2_O), 90 mg; Se (Na_2_SeO_3_), 0.3 mg.

3 Nutrient levels of crude protein, crude fiber, calcium, and phosphorus were analyzed, and others were calculated according to the Feed Composition and Nutritive Values in China (China Feed Database, 2020).

Sows received 2.5 kg/d of experimental diets from 90 d of gestation until farrowing. On the day of farrowing, sows were not fed. From the following day onward, the feed supply was increased by 1 kg/d until d 3 and then by 0.5 kg/d until d 6. Afterward, sows were allowed to feed ad libitum until d 21 of lactation. The experiment began from d 90 of gestation and ended on d 21 of lactation (d 21 postpartum), when piglets were weaned and sows were relocated to the breeding facility. At d 21 postpartum, 24 piglets (one per litter; 5.32 ± 0.07 kg BW) were assigned to a 2 × 2 factorial arrangement of sow dietary treatment (2% SO vs. 2% FABO) and lipopolysaccharide (LPS) challenge (saline vs. LPS). All piglets were grouped by sow dietary treatment. Within each maternal diet group, half of the piglets received an intraperitoneal injection of either 100 μg/kg BW of LPS (*Escherichia. coli* serotype O55:B5, Sigma Chemical Inc., St. Louis, MO, USA) or equivalent sterile saline. The dose of LPS was chosen based on previous study (Liu et al., 2012).

Sows were housed individually in pens (2.2 m × 1.5 m) throughout the trial. On d 107 of gestation, the sows were moved to the farrowing room. The temperature in the farrowing room was maintained between 23 and 26 °C.

### 2.3 Sample collection

The feed intake of sows was recorded throughout lactation to calculate average daily feed intake (ADFI). The BF thickness was measured at the P2 position (6 cm to the left side of the dorsal midline at the head of the last rib) using an ultrasonic device (Lean-Meater, Renco Corp., Golden Valley, MN, USA) and recorded on d 90 of gestation, within 24 h after farrowing and at weaning to calculate lactation BF loss. At parturition, litter size data (total, live-born, and stillborn) were collected. Litter and individual piglet birth weight were recorded. Litters were then standardized to 10 to 11 piglets per sow through cross-fostering within the same dietary group. Piglet numbers and body weights were recorded after cross-fostering and at weaning.

Within 2 h after the birth of the first piglet, 30 mL of colostrum was collected manually from all functional glands per sow. A 10 mL aliquot of colostrum was centrifuged (4000 × *g* for 15 min, at 4 °C), and then the supernatant was harvested and stored at −80 °C for subsequent immunoglobulins (immunoglobulin [Ig] G, IgM, and IgA) analysis. The remaining 20 mL portion was kept at −20 °C for general composition analysis.

At 4 h post-injection, piglets were euthanized and the intestine was promptly excised. A 2 cm segment from the mid-jejunum was fixed in 4% paraformaldehyde for histology. An adjacent 15 cm segment was opened longitudinally, flushed with ice-cold phosphate-buffered saline. Mucosal samples were scraped off, snap-frozen in liquid nitrogen, and then stored at −80 °C for subsequent analysis.

### 2.4 Chemical analysis

The colostral composition was measured by an automatic milk analyzer (Lacto Scope 200 FT-IR, PerkinElmer, Inc., Waltham, MA, USA). The levels of IgG (RX2D711366), IgM (RX2D711436), and IgA (RX2D714286) in the colostrum supernatant were measured by competitive enzyme-linked immunosorbent assay (ELISA) method using swine commercial kits (Quanzhou Ruixin Biotech Co., Ltd., Quanzhou, Fujian, China) according to the manufacturer's instructions. The intra- and inter-assay coefficients of variation were less than 10%, 10% ,and 15% respectively.

Approximately 0.3 g of frozen jejunal mucosal sample from piglet was added to 2.7 mL of ice saline, homogenized, and then centrifuged at 12,000 × *g* for 5 min at 4 °C. Then, the supernatant was collected. The levels of secretory immunoglobulin A (sIgA) in jejunal mucosa were detected using porcine commercial ELISA kits (RX500383P, Quanzhou Ruixin Biotech Co., Ltd., Quanzhou, Fujian, China). The jejunal disaccharidase (sucrase [A082-2-1], maltase [A082-3-1], and lactase [A082-1-1]) activities were determined with commercial kits (Nanjing Jiancheng Bioengineering Institute, Nanjing, Jiangsu, China) using a microplate reader (iMark, Bio-Rad Laboratories, Inc., Hercules, CA, USA) according to the manufacturer's instructions.

### 2.5 Intestinal histomorphology and histology

The fixed jejunal tissue was embedded in paraffin, sectioned in to 4 μm slices, and then stained with hematoxylin and eosin. The slices were observed under a microscope. The ten villous height and their associated crypts from each slice were measured. Jejunal goblet cells were stained with Alcian blue and periodic acid-Schiff (AB/PAS) commercial kit (G1285, Beijing Solarbio Bioscience Technology Co., Ltd., Beijing, China), following the manufacturer's instructions. The goblet cells were counted under a microscope (BX5, Olympus Corp., Tokyo, Japan) using a 200 × objective, and the density of goblet cell was calculated from 10 randomly selected crypt-villi per slice.

### 2.6 Western blot analysis

The total protein of jejunal mucosa was harvested using radioimmunoprecipitation assay (RIPA) buffer with phenylmethane sulfonyl fluoride (P0013C, Beyotime Biotechnology Inc., Shanghai, China). The content of protein was determined by bicinchoninic acid (BCA) protein assay kit (P0010S, Beyotime Biotechnology Inc., Shanghai, China) according to the manufacturer's protocol. The proteins were separated by polyacrylamide gel electrophoresis and transferred to polyvinyl difluoride membranes. After blocking with 5% skim milk for 2 h at room temperature, membranes were incubated with the primary antibody and appropriate secondary antibodies. The primary antibodies included anti-claudin-1 (71-7800, 1:1000, Thermo Fisher Scientific Inc., Waltham, MA, USA), anti-ZO-1 (abs122482, 1:1000, Absin Bioscience Inc., Shanghai, China), anti-IL-22 (ab181007, 1:1000, Abcam PLC, Cambridge, UK), anti-STAT3 (bs55208R, 1:1000, Beijing Bioss Biotechnology Co., Ltd., Beijing, China), anti-pSTAT3 (bsm52210R, 1:1000, Beijing Bioss Biotechnology Co., Ltd., Beijing, China), anti-β-actin (ZMS1156, 1:1000, Sigma-Aldrich Inc., St. Louis, MO, USA). The full name of the abbreviations of all proteins can be found in Table S1. Subsequently, the protein bands were detected with an enhanced Chemiluminescence Western blotting kit (Amersham Biosciences Co., Uppsala, Sweden). The band densities were analyzed with ImageJ software (version 4.3.17; Synoptics Ltd., Frederick, MD, USA) and normalized to that of β-actin.

### 2.7 Quantitative real-time PCR for gene expression analysis

Total RNA from jejunal mucosa was isolated with TRIzol Reagent (9108, TaKaRa Biotechnology Co., Ltd., Dalian, Liaoning, China) according to the manufacturer's protocols. Total RNA (2 μg) was used to synthesize complementary DNA (cDNA) with PrimeScript RT reagent kit with genomic DNA (gDNA) eraser (RR047A, TaKaRa Biotechnology Co., Ltd., Dalian, Liaoning, China). Quantitative real-time PCR analyses were conducted on the Applied Biosystems 7500 Real-time PCR System (Thermo Fisher Scientific Inc., Waltham, MA, USA) using a SYBR Premix Ex Taq (Tli RNase H Plus) qPCR kit (RR420A, TaKaRa Biotechnology Co., Ltd., Dalian, Liaoning, China). The primer sequences are presented in Table S2. The full names of the abbreviations of all genes can be found in Table S1. The quantitative real-time PCR reaction conditions were as follows: the first denaturation (95 °C for 30 s), 40 cycles of denaturation (95 °C for 5 s), annealing (60 °C for 30 s), extension (72 °C for 34 s), and a final extension (72 °C for 5 min). Relative changes in gene expression were calculated using the 2^−ΔΔCt^ method (Livak and Schmittgen, 2001) and β-actin was used as a reference gene.

### 2.8 Statistical analysis

All data are presented as the mean values with their standard errors (SEM). The data of sow reproductive performance, colostrum composition, and IgG, IgM, and IgA levels were analyzed using an independent-sample *t*-test (SPSS 22.0, IBM, Armonk, NY, USA). Other data were evaluated as a 2 × 2 factorial design using the general linear model (GLM) procedures of SPSS 22.0. The statistical model included the effects of sow diet (2% SO or 2% FABO), LPS challenge (LPS or saline), and their interaction. When there was a significant or trend interaction between sow diet and LPS challenge, post hoc testing was performed using Duncan's multiple comparison tests. *P* < 0.05 is considered statistically significant, and 0.05 ≤ *P* ≤ 0.10 is considered a trend toward significance. The mathematical model was as follows:

$$Y_{ij}=\mu +\alpha_{i}+\beta_{j}+{\left(\alpha \beta \right)}_{ij}+\varepsilon_{ijk}\text{,}$$

where *Y*_*ij*_ is the observation; *μ* is the general mean; *α*_*i*_ is the effect of LPS challenge; *β*_*j*_ is the sow dietary treatment; (αβ)_*ij*_ is the interaction between the LPS challenge and the sow dietary treatment; ε_*ijk*_ is the random error.

## 3 Results

### 3.1 Performance of sows and piglets

Table 3 presents the performance of the sow and piglets. Compared to 2% SO group, dietary supplementation with 2% FABO had no effect on sows BF loss or ADFI (*P* > 0.05). Moreover, total piglet born count and born alive, litter birth weight, or weaning weight did not differ between 2% SO and 2% FABO fed sows (*P* > 0.05). However, maternal dietary 2% FABO supplementation increased the piglet birth weight and weaning litter weight compared with 2% SO group (*P* < 0.05). The litter weight gain and ADG of piglets from 2% FABO sows were also greater than those from 2% SO sows (*P* < 0.05). In addition, weaning survivability tended to improve in offspring from FABO-fed sows compared to those from SO-fed sows (*P* = 0.076).

**Table 3:** Effects of dietary supplementation with FABO on sow reproductive and piglet growth performance.

Items	Groups^2^		SEM	*P*-value
	2% SO	2% FABO		
**Sows**				
Parity	2.25	2.33	0.353	0.909
BF thickness at d 90, mm	17.23	16.99	0.525	0.822
BF thickness at farrowing, mm	18.45	17.58	0.511	0.412
BF thickness at weaning, mm	13.71	13.58	0.649	0.926
BF loss during lactation, mm	4.75	3.99	0.500	0.471
ADFI^1^, kg/d	4.16	3.87	0.117	0.219
**Piglets**				
Total born	13.27	13.25	0.561	0.985
Born alive, %	97.97	98.11	0.910	0.945
Litter size after cross-foster	11.17	11.00	0.268	0.764
Litter size at weaning	9.92	10.58	0.254	0.226
Weaning survivability, %	89.34	96.47	1.998	0.076
Litter birth weight, kg	18.93	20.78	0.711	0.214
Pig birth weight, kg	1.42^b^	1.57^a^	0.033	0.018
Pig weights after cross-foster, kg	1.45^b^	1.56^a^	0.016	0.001
Weaning litter weight, kg	56.02^b^	66.83^a^	2.350	0.017
Pig weaning weight, kg	5.75	6.22	0.159	0.147
ADG, g/d	197.00^b^	224.50^a^	5.700	0.012
Litter weight gain, kg	39.38^b^	50.45^a^	2.787	0.044

ADFI  =  average daily feed intake; ADG  =  average daily gain; BF  =  backfat; SO  =  soybean oil; FABO  =  fatty acid-balanced oil; SEM  =  standard error of the mean.

Means in a row with different superscripts differ significantly (*P* < 0.05), *n* = 12.

1 ADFI was recorded from farrowing until 21 d postpartum.

2 SO, diet supplemented with 2% soybean oil group; FABO, diet supplemented with 2% fatty acid-balanced oil group.

### 3.2 Colostrum composition and IgG, IgA, and IgM concentration

The composition of colostrum is shown in Table 4. The FABO group exhibited elevated colostral protein, IgG, IgM, and IgA concentrations compared to SO-fed sows (*P* < 0.05). In contrast, fat and lactose contents of colostrum did not differ between dietary treatments (*P* > 0.05).

**Table 4:** Effects of dietary supplementation with FABO on the chemical composition of colostrum.

Items	Groups^1^		SEM	*P-*value
	2% SO	2% FABO		
Fat, %	4.47	4.66	0.408	0.826
Protein, %	14.17^b^	15.40^a^	0.296	0.043
Lactose, %	2.15	2.03	0.095	0.519
Solid-non-fat, %	17.31	17.54	0.266	0.680
IgG, g/L	27.51^b^	32.68^a^	1.260	0.036
IgM, g/L	4.58^b^	5.62^a^	0.249	0.031
IgA, g/L	0.75^b^	1.51^a^	0.108	<0.001

SO = soybean oil; FABO = fatty acid-balanced oil; Ig = immunoglobulin; SEM  =  standard error of the mean.

Means in a row with different superscripts differ significantly (*P* < 0.05), *n* = 12.

1 SO, diet supplemented with 2% soybean oil group; FABO, diet supplemented with 2% fatty acid-balanced oil group.

### 3.3 Intestinal morphology

Lipopolysaccharide challenge caused intestinal mucosal injury, characterized by epithelial lifting at the tip of the villus and villous atrophy (Fig. 1), which was partially mitigated in piglets suckling 2% FABO-supplemented sows. The LPS administration decreased jejunal villus height in piglets compared with the saline-treated group (*P* = 0.002; Table 5). Piglets suckling 2% FABO-supplemented sows had higher villus height in the jejunum than those suckling 2% SO-supplemented sows (*P* = 0.029). An interactive trend was observed between sow dietary treatment and LPS challenge for crypt depth in piglet jejunum (*P* = 0.092). Maternal dietary 2% FABO supplementation increased crypt depth in piglets treated with saline (*P* = 0.026), whereas there was no difference among LPS-treated piglets (*P* > 0.05).

**Fig. 1:**
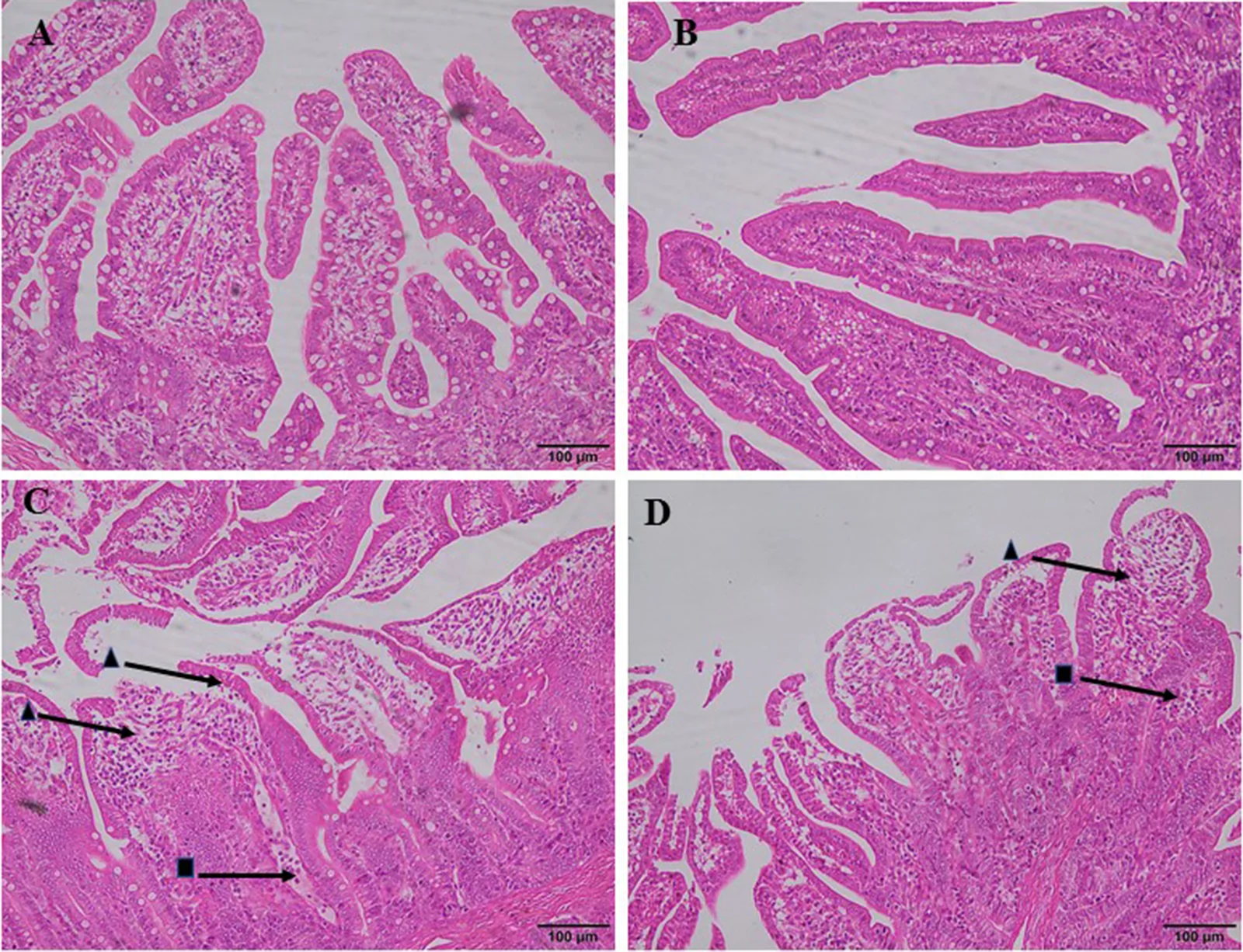
Effects of maternal supplementation fatty acid-balanced oil (FABO) on intestinal morphology in lipopolysaccharide (LPS)-challenged piglets. (A) 2% SO group, piglets from sows fed a diet supplemented with 2% soybean oil and injected with saline. (B) 2% FABO group, piglets from sows fed a diet supplemented with 2% fatty acid-balanced oil and injected with saline. (C) 2% SO + LPS group, piglets from sows fed a diet supplemented with 2% soybean oil and injected with LPS. (D) 2% FABO + LPS group, piglets from sows fed a diet supplemented with 2% fatty acid-balanced oil and injected with LPS. Jejunum with hematoxylin and eosin staining (original magnification 200×, scale bar = 100 μm), indicates lifting of epithelium at the tip of the villus and indicates villous atrophy. SO = soybean oil.

**Table 5:** Effects of maternal supplementation FABO on intestinal morphology in lipopolysaccharide (LPS)-challenged piglets^1^.

Items	Saline		LPS		SEM	*P-*value		
	2% SO	2% FABO	2% SO	2% FABO		Diet	LPS	Diet × LPS
Villus height, μm	222.58	250.99	201.74	212.69	4.196	0.029	0.002	0.311
Crypt depth, μm	100.13^b^	121.89^a^	100.98^b^	99.34^b^	3.305	0.144	0.116	0.092
Villus height/crypt depth	2.29	2.13	2.08	2.19	0.043	0.774	0.393	0.114

SO = soybean oil; FABO = fatty acid-balanced oil; SEM  =  standard error of the mean.

Means in a row with different superscripts differ significantly among treatments (*P* < 0.05, Duncan's multiple range test was used when interaction effects were significant or showed a significant trend), *n* = 6.

1 SO, diet supplemented with 2% soybean oil group; FABO, diet supplemented with 2% fatty acid-balanced oil group.

### 3.4 The activities of disaccharidase in piglet jejunum

Piglets challenged with LPS had lower jejunal sucrase activity than the saline-treated group (*P* < 0.001; Table 6). A sow dietary treatment and LPS challenge interaction was detected for maltase and sucrase activities (*P* < 0.05). Among LPS-treated piglets, sow dietary 2% FABO supplementation increased maltase activity (*P* = 0.001), whereas there was no difference among saline-treated piglets (*P* > 0.05). Maternal dietary 2% FABO supplementation increased sucrase activity in piglets treated with LPS (*P <* 0.001), while 2% FABO supplementation increased sucrase activity in saline-treated piglets (*P* = 0.015). Neither LPS challenge nor maternal dietary treatment affected jejunal lactase activity in piglet (*P* > 0.05).

**Table 6:** Effects of maternal supplementation FABO on jejunal disaccharidase activity (U/mg protein) in lipopolysaccharide (LPS)-challenged piglets^1^.

Items	Saline		LPS		SEM	*P-*value		
	2% SO	2% FABO	2% SO	2% FABO		Diet	LPS	Diet × LPS
Sucrase	162.07^a^	131.51^b^	71.69^c^	145.78^ab^	4.078	0.015	<0.001	<0.001
Maltase	237.55^ab^	231.79^b^	198.55^b^	280.60^a^	7.652	0.022	0.752	0.009
Lactase	343.46	345.38	323.88	374.02	8.665	0.148	0.796	0.179

SO = soybean oil; FABO = fatty acid-balanced oil; SEM  =  standard error of the mean.

Means in a row with different superscripts differ significantly among treatments (*P* < 0.05, Duncan's multiple range test was used when interaction effects were significant or showed a significant trend), *n* = 6.

1 SO, diet supplemented with 2% soybean oil group; FABO, diet supplemented with 2% fatty acid-balanced oil group.

### 3.5 Intestinal mucosa barrier

Jejunal goblet cell number was reduced in LPS-challenged piglets compared with saline-treated piglets (*P* < 0.001; Fig. 2A and B). There was an interaction between sow dietary treatment and LPS challenge on jejunal goblet cell number (*P* = 0.048). Maternal dietary 2% FABO supplementation increased goblet cell number in piglets treated with LPS (*P* < 0.001), whereas there was no difference among saline-treated piglets (*P* > 0.05).

**Fig. 2:**
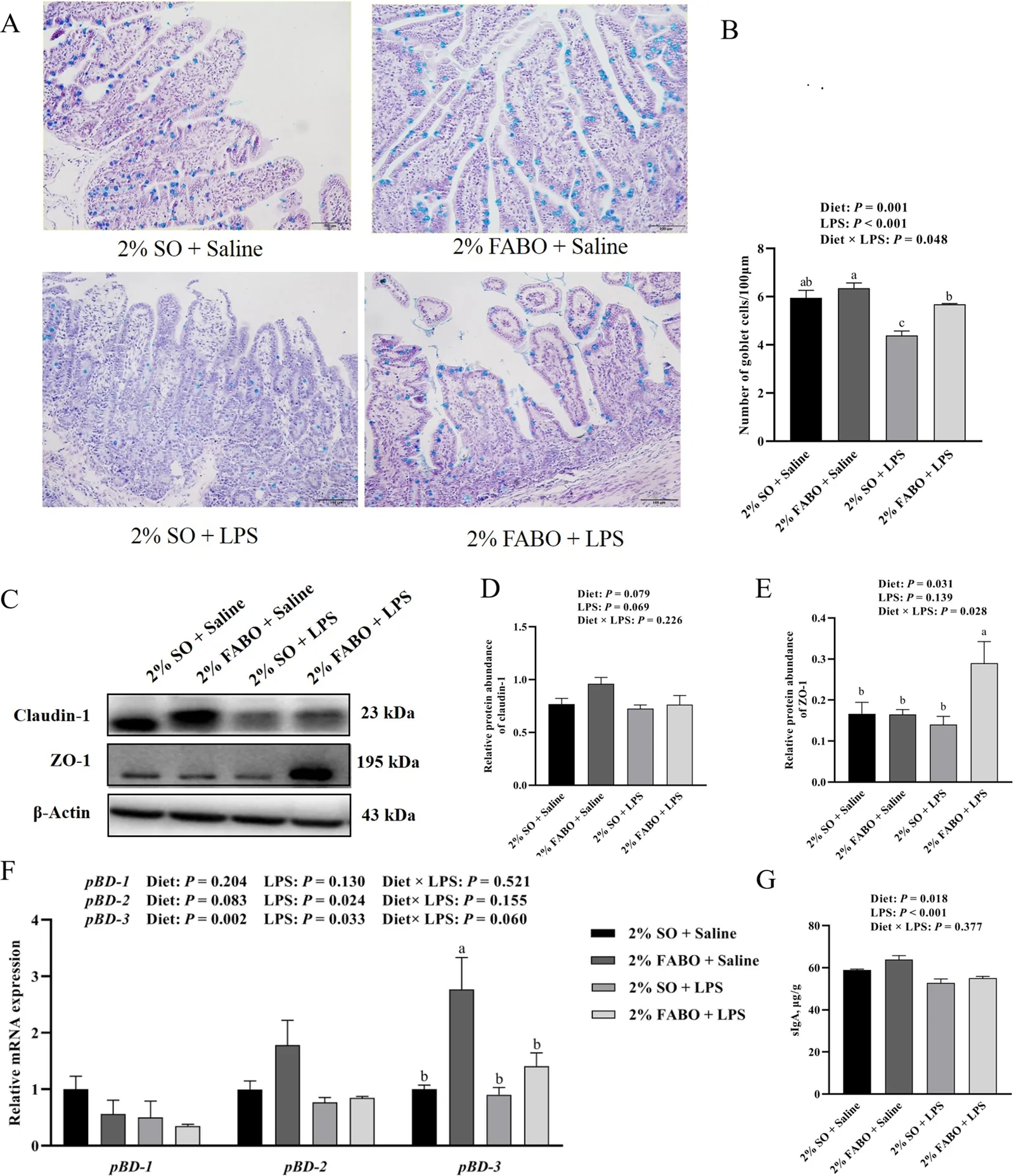
Effects of maternal supplementation fatty acid-balanced oil (FABO) on intestinal mucosal barrier in lipopolysaccharide (EPS)-challenged piglets. 2% SO, piglets from sows fed a diet supplemented with 2% soybean oil and injected with saline; 2% FABO, piglets from sows fed a diet supplemented with 2% fatty acid-balanced oil and injected with saline; 2% SO + LPS, piglets from sows fed a diet supplemented with 2% soybean oil and injected with LPS; 2% FABO + LPS, piglets from sows fed a diet supplemented with 2% fatty acid-balanced oil and injected with LPS. (A) Periodic acid Schiff (PAS)-stain pictures of the jejunum (original magnification 200×, scale bar = 100 μm). (B) The number of goblet cells. (C–E) Jejunal tight junction protein (claudin-1 and ZO-1) expression. (F) Jejunal *pBD-1*, *pBD-2* and *pBD-*3 mRNA expression. (G) Concentration of sIgA in the jejunum. SO = soybean oil; sIgA = secretory immunoglobulin A. Different lowercase letters above columns represent significant differences among treatments at *P* < 0.05 (Duncan's multiple range test was used when interaction effects were significant or showed a significant trend, *n* = 6).

Lipopolysaccharide administration tended to decrease the expression of jejunal claudin-1 in piglets compared with the saline-treated group (*P* = 0.069; Fig. 2C and D). Piglets suckling 2% FABO-supplemented sows tended to have higher protein expression of claudin-1 compared with those suckling 2% SO-supplemented sows (*P* = 0.079). There was a maternal dietary treatment × LPS challenge interaction observed for protein expression of ZO-1 in the jejunum of piglets (*P* = 0.028; Fig. 2C and E). Sows dietary 2% FABO supplementation increased the protein expression of ZO-1 in piglets treated with LPS (*P* = 0.003), whereas there was no difference among saline-treated piglets (*P* > 0.05).

Lipopolysaccharide challenge reduced mRNA abundance of *pBD-2* and *pBD-3* in the jejunum compared with the saline-treated group (*P* < 0.05; Fig. 2F). Piglets suckling 2% FABO-supplemented sows tended to increase *pBD-*2 mRNA abundance in the jejunum compared with those suckling 2% SO-supplemented sows (*P* = 0.083). There was a trend toward an interaction between sow dietary treatment and LPS challenge observed for *pBD-*3 mRNA abundance in the piglet jejunum (*P* = 0.060). Maternal dietary 2% FABO supplementation increase mRNA abundance of *pBD-3* in saline-treated piglets (*P* = 0.001), whereas there was no difference in LPS-treated piglets (*P* > 0.05).

Lipopolysaccharide challenge decreased the concentration of jejunal sIgA in piglets compared with the saline-treated group (*P* < 0.001; Fig. 2G). Compared to piglets from 2% SO-fed sows, piglets from 2% FABO-fed sows had higher sIgA level in the jejunum (*P* = 0.018). There was no significant dietary treatment and LPS challenged interaction observed for jejunal sIgA concentration (*P* = 0.377).

### 3.6 The mRNA level of inflammatory factor relative genes in the jejunum of piglets

Administration of LPS enhanced *IL-1β*, *HSP-70*, and *COX2* mRNA abundance (*P* < 0.05) and tended to elevate *IL-*6 mRNA abundance (*P* = 0.080) in piglet jejunum compared with the saline-treated group (Fig. 3). Compared with piglets suckling 2% SO-supplemented sows, piglets suckling 2% FABO-supplemented sows had lower mRNA abundance of *HSP-70* in the jejunum (*P* = 0.044). Moreover, sow dietary 2% FABO supplementation enhanced *IL-*10 mRNA abundance (*P* = 0.001) and tended to enhance *COX2* mRNA expression (*P* = 0.085) in piglets compared with sow dietary 2% SO supplementation. There was a sow dietary treatment and LPS challenge interaction for *IL-*6 mRNA abundance (*P* = 0.001) and a trend toward interaction for *IL-1β* mRNA abundance (*P* = 0.091). Maternal dietary 2% FABO supplementation decreased mRNA abundance of *IL-6* (*P* = 0.001) in piglets treated with LPS, whereas there was no difference among saline treated piglets (*P* > 0.05). Lipopolysaccharide challenged did not affected mRNA abundance of *IL-1β* in piglets suckling 2% FABO-supplemented sows (*P* > 0.05), while 2% SO increased mRNA abundance of *IL-1β* in LPS-treated piglets compared to saline-treated piglets (*P* = 0.010).

**Fig. 3:**
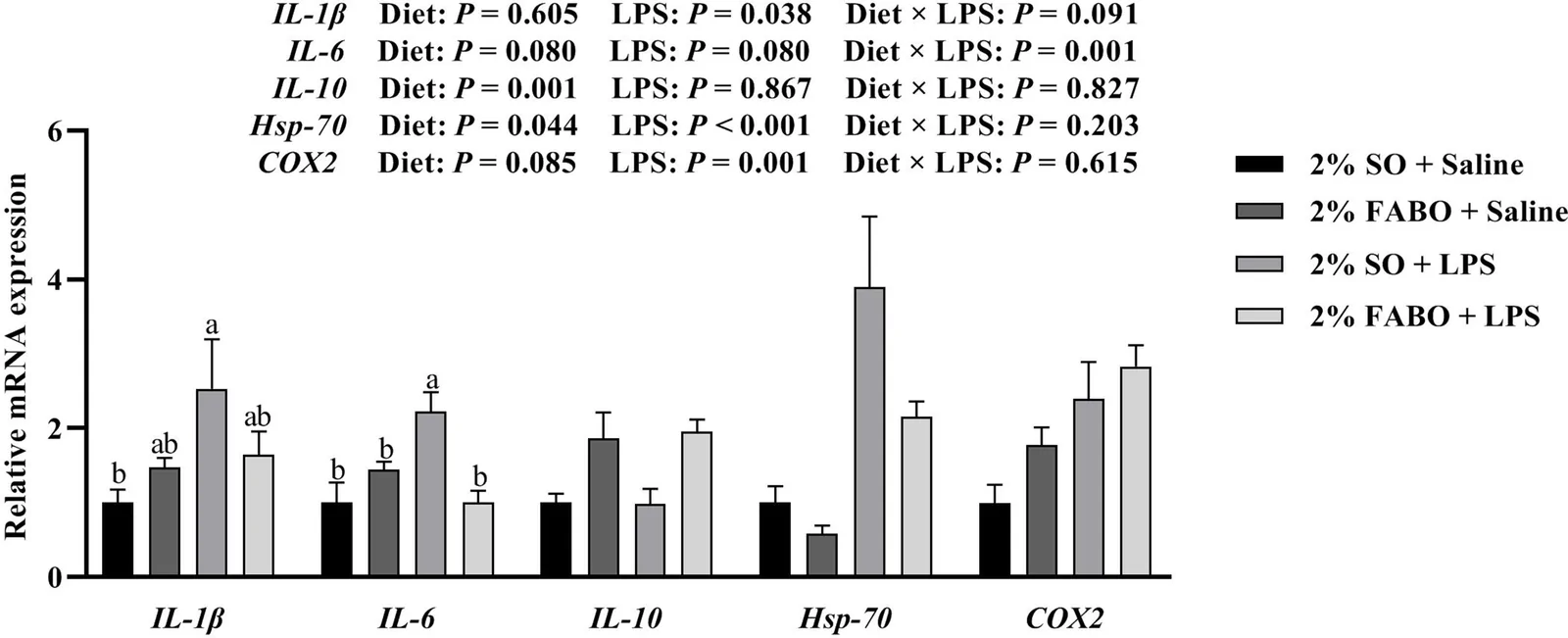
Effect of maternal supplementation fatty acid-balanced oil (FABO) on inflammatory factor mRNA expression in the jejunum of lipopolysaccharide-challenged piglets. 2% SO, piglets from sows fed a diet supplemented with 2% soybean oil and injected with saline; 2% FABO, piglets from sows fed a diet supplemented with 2% fatty acid-balanced oil and injected with saline; 2% SO + LPS, piglets from sows fed a diet supplemented with 2% soybean oil and injected with LPS; 2% FABO + LPS, piglets from sows fed a diet supplemented with 2% fatty acid-balanced oil and injected with LPS. SO = soybean oil. Different lowercase letters above columns represent significant differences among treatments at *P* < 0.05 (Duncan's multiple range test was used when interaction effects were significant or showed a significant trend, *n* = 6).

### 3.7 Gene and protein expression related to IL-22/STAT3 pathway in jejunum of piglets

Lipopolysaccharide challenge reduced mRNA abundance of *TYK2* (*P* = 0.049), tended to decrease mRNA abundance of *IL-22* (*P* = 0.088) and increase *IL-22R1* mRNA abundance (*P* = 0.086) in piglet jejunum compared with saline-treated group (Fig. 4A). Compared with piglets suckling 2% SO-supplemented sows, piglets suckling 2% FABO-supplemented sows had higher mRNA abundance of *TYK2* and *STAT3* (*P* < 0.05), and tended to have higher *IL-*22 mRNA abundance (*P* = 0.077) in the jejunum. There was a sow dietary treatment × LPS challenge interaction observed for *JAK1* mRNA abundance (*P* = 0.023). Maternal dietary 2% FABO supplementation enhanced mRNA abundance of *JAK1* in piglets treated with saline (*P* < 0.001), whereas there was no difference among LPS-treated piglets (*P* > 0.05). Furthermore, piglets challenged with LPS tended to decrease IL-22 protein expression in the jejunum compared with those treated with saline (*P* = 0.053; Fig. 4B and C). Sow dietary 2% FABO supplementation enhanced IL-22 protein expression (*P* = 0.030). There was a sow dietary treatment × LPS challenge interaction for p-STAT3 protein expression (*P* = 0.013; Fig. 4B–E) and a trend toward interaction for p-STAT3:t-STAT3 ratio (*P* = 0.060). Maternal dietary 2% FABO supplementation increased p-STAT3 protein expression (*P* = 0.007) in piglets treated with LPS, whereas there was no difference among saline treated piglets (*P* > 0.05). Lipopolysaccharide challenge increased p-STAT3:t-STAT3 ratio in piglets suckling 2% FABO-supplemented sows (*P* = 0.014), whereas 2% SO supplementation did not affect p-STAT3:t-STAT3 ratio (*P* > 0.05).

**Fig. 4:**
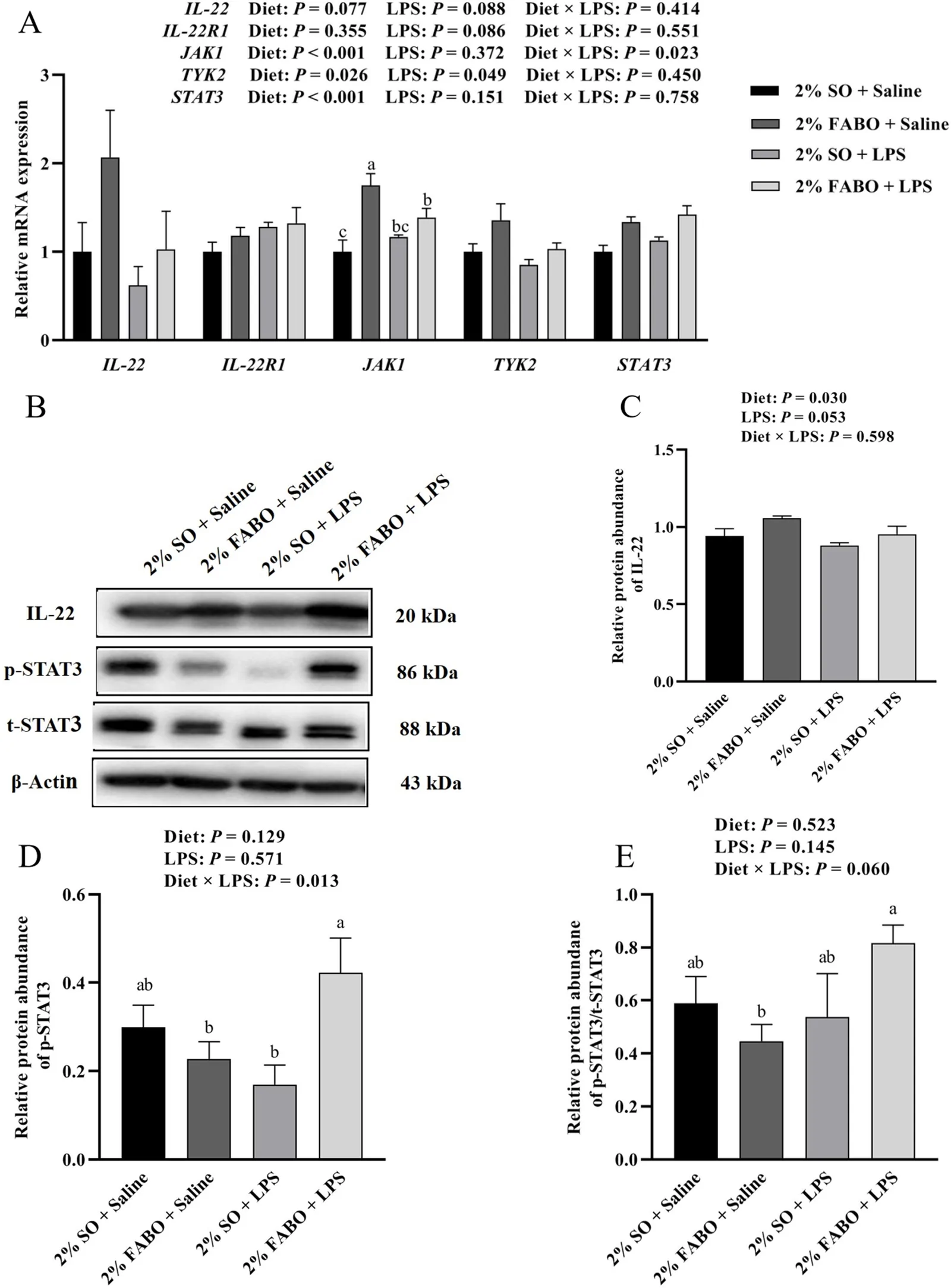
Effect of maternal supplementation fatty acid-balanced oil (FABO) on the IL-22/STAT3 signaling pathway in the jejunum of lipopolysaccharide-challenged piglets. (A) The relative mRNA expression level of *IL-22*, *IL-22R1*, *JAK1*, *TYK2*, and *STAT3* in the jejunum. (B) Representative bands of IL-22, t-STAT3, p-STAT3, and β-actin in the jejunum. (C–E) The relative protein expression of IL-22, p-STAT3 and p-STAT3/t-STAT3. 2% SO, piglets from sows fed a diet supplemented with 2% soybean oil and injected with saline; 2% FABO, piglets from sows fed a diet supplemented with 2% fatty acid-balanced oil and injected with saline; 2% SO + LPS, piglets from sows fed a diet supplemented with 2% soybean oil and injected with LPS; 2% FABO + LPS, piglets from sows fed a diet supplemented with 2% fatty acid-balanced oil and injected with LPS. SO = soybean oil. Different lowercase letters above columns represent significant differences among treatments at *P* < 0.05 (Duncan's multiple range test was used when interaction effects were significant or showed a significant trend, *n* = 6).

## 4 Discussion

Maternal nutrition is fundamental to optimal fetal development and postnatal offspring growth (Emamverdi et al., 2019; Hambidge and Krebs, 2018). Sows undergo complex metabolism and immune changes to meet offspring nutritional and energetic demands during late gestation and lactation (Quesnel et al., 2009; Wang et al., 2022). Notably, insufficient energy intake constrains sow reproductive performance and directly compromises piglet health. Evidence indicated that fat supplementation during late gestation and lactation could improve both sow reproductive performance and litter growth performance (Jin et al., 2017; Wang et al., 2022). However, most of the studies have focused on individual fats or fatty acids. Studies on FABO remain predominantly confined to humans and mice, with limited data available for swine. Therefore, the objective of this study was to evaluate the effects of maternal dietary FBAO supplementation from d 90 of gestation until d 21 postpartum on both the productive performance of sows and offspring growth. Furthermore, this study sought to evaluate whether maternal FABO supplementation could mitigate LPS-induced intestinal barrier damage in nursing piglets.

In the current study, there was no difference in sow BF thickness at either farrowing or weaning between dietary treatments. Previous studies have demonstrated that supplementation with different fat or fatty acid sources in the sow diet did not affect BF thickness loss (Gatlin et al., 2002; Jang et al., 2017; Sun and Kim, 2020). Furthermore, FABO (a product of mixing various oils and fats) supplementation did not influence sow reproductive performance (Zhang et al., 2024). Relative BF loss is a key indicator of energy mobilization status in sows. In the present study, the absence of dietary treatment effects on BF loss might be explained by the isoenergetic design of the experimental diets.

Owing to the epitheliochorial placenta that restricts transplacental transfer, neonatal piglets possess minimal energy reserve and lack innate immune protection (Rooke and Bland, 2002; Salmon et al., 2009). In addition, the immune system of newborn piglets is not fully developed and consequently depends on maternally derived passive immunity. As the primary postnatal resource, colostrum contains energy-rich nutrients, maternal Ig, and bioactive growth factors, which are essential for piglets survival and growth. In this study, fat content in colostrum was not influenced by sow dietary FABO supplementation. In agreement with the results of this study, Jang et al. (2017) found that dietary sodium butyrate supplementation did not alter colostral fat content in sows. Indeed, colostral fat and lactose contents rise alongside dietary energy density in sows over late gestation and lactation (Che et al., 2019; Heo et al., 2008). In this study, FABO supplementation did not alter fat content in colostrum, which was associated with similar energy intake of sows. In addition, the result of this study showed that colostral protein content was elevated in FABO-supplemented sows. This aligns with Chen et al. (2019), who reported increased colostral protein after supplementing sodium butyrate, medium-chain fatty acids and n-3 PUFA. Conversely, Jin et al. (2017) indicated that protein content in sow colostrum and milk remained unaffected by fat source (palm oil, fish oil, or soybean oil). Currently, the mechanistic basis for dietary fat or fatty acid sources on sow colostrum protein content remains to be established. It was speculated that FABO-associated protein enrichment might reflect enhanced Ig transfer. Colostral Ig constitute the sole source of passive immunity for neonatal piglets. Moreover, sow milk Ig concentrations are modulated by dietary fatty acid composition. Chen et al. (2019) demonstrated that sow dietary supplementation with sodium butyrate, medium-chain and n-3 PUFAs elevated IgG, IgM, and IgA levels in colostrum. Combined sodium butyrate, medium-chain fatty acids and n-3 PUFAs in sow diets enhanced colostral IgG, IgA, and IgM contents (You et al., 2023). Similarly, the results of this study showed that feeding sows with FABO increased the contents of IgG, IgM, and IgA in colostrum. The mechanism by which dietary FABO supplementation increased mammary Ig levels in sows remained unclear. A possible explanation is that n-3 PUFAs promote mammary epithelial cell growth and differentiation, thereby improving lactation performance (Li et al., 2025). In addition, n-3 and n-6 PUFA enhance IL-2 synthesis, which in turn stimulates the production of IgG, IgM, and IgA (Kawano and Noma, 1996; Yao et al., 2012). In the present study, the increase in colostral Ig levels might be attributed to the synergistic effect of the various fatty acids in FABO.

Milk composition directly impacts neonatal health and development (Gao et al., 2023). Jin et al. (2017) reported that dietary fish oil supplementation in sows increased IgG and IgM levels in colostrum and milk, while also improving piglet performance such as litter weaning weight, weaning survival rate and total litter weight gain. Similarly, the results of this study showed that offspring from FABO-supplemented sows exhibited improved weaning survival rate, weaning litter weight, litter weight gain, and ADG. These enhancements likely reflected elevated colostral protein and Ig (IgG, IgM, and IgA) concentrations.

The intestine is the primary site for nutrient digestion and absorption, while also serving as a complex defense barrier (Furness et al., 2013; Ren et al., 2020). Compromise of intestinal barrier function facilitates the invasion of bacteria and endotoxins, leading to in intestinal inflammation, ultimately reducing piglet growth performance (Zhao et al., 2021). Previous studies have demonstrated that maternal nutrition influenced offspring intestinal development and function (Desaldeleer et al., 2014; Li et al., 2021). The intestinal morphology is associated with intestinal health and growth performance in pigs (Walker, 1959). In the present study, LPS challenge induced significant morphological injury to the intestinal mucosa, including epithelial exfoliation of the villus and villus height reduction. Maternal FABO supplementation mitigated intestinal injury induced by LPS in offspring, as evidenced by enhanced jejunal villus height. Consistent with the results of this study, Chwen et al. (2013) reported that maternal supplementation with medium-chain triglycerides increased jejunal villus height in suckling piglets. The elevated villus height suggests an expanded absorptive surface area, thereby improving nutrient utilization and growth performance. In addition, the activity of mucosal disaccharidases is a widely recognized marker of enterocyte functional maturation, reflecting the digestive competence of the intestine (Pi et al., 2014). Takase and Goda (1990) reported that feeding medium-chain triglycerides increased sucrase activities in the rat small intestine. Similarly, in the present study, sow FABO supplementation increased maltase and sucrase activities in the offspring's jejunum. Moreover, in the FABO group, piglets challenged with LPS exhibited higher jejunal sucrase and maltase activities than those not challenged. A possible explanation is that maternal FABO supplementation preserved villus structure and promoted intestinal maturation of offspring. Thus, enterocytes might exhibit a compensatory response upon LPS challenge, increasing enzyme activity to maintain digestive function.

Tight junction proteins (ZO-1, occludin, and claudin-1) orchestrate intestinal architecture and barrier integrity (Liao et al., 2017). Reduced tight junction protein expression impairs intestinal barrier integrity. In the present study, LPS challenge tended to reduce the expression of claudin-1, while maternal FABO supplementation increased jejunal tight junction proteins (claudin-1 and ZO-1) expression in offspring. Similarly, Chen et al. (2019) indicated that maternal sodium butyrate, medium-chain fatty acids or n-3 PUFA upregulated mRNA expression of jejunal claudin-1 and ZO-1 in suckling piglets. Cytokines critically regulate both innate immunity and the integrity of the intestinal barrier (Liao et al., 2020). Pro-inflammatory cytokines enhance the permeability of the intestinal barrier, whereas anti-inflammatory cytokines reinforce the intestinal barrier function (Ji et al., 2022). Sow FABO supplementation decreased the mRNA expression of *HSP-70* and *IL-6*, and increased anti-inflammatory *IL-10* expression in the offspring jejunum. Liu et al. (2012) reported that dietary fish oil supplementation decreased the TNF-α concentration and *HSP-70* expression in the jejunum of pigs. Chen et al. (2019) demonstrated that maternal dietary supplementation with fatty acids (sodium butyrate, medium-chain fatty acids, or n-3 PUFA) decreased *IL-1β* and *TNF-α* mRNA expression in the jejunum of offspring by inhibiting TLR4 signaling. Moreover, n-3 PUFAs activate PPARγ, which in turn suppresses pro-inflammatory cytokine expression (Huang et al., 2015). These findings suggest that maternal dietary supplementation with FABO may alleviate inflammation, and consequently improve intestinal barrier integrity in offspring.

The intestinal mucosal surface comprises a protective glycocalyx enriched with mucins, antimicrobial peptides (AMPs; defensins and C-type lectins), and sIgA, which prevents pathogen adhesion and epithelial translocation (Johansson et al., 2013). Mucin synthesis occurs predominantly in goblet cells. Goblet cell dysfunction or numeric aberrations heighten susceptibility to bacterial invasion and mechanical insult (Mahapatro et al., 2016). The AMPs confer protection against invading pathogens and promote mucin production in the intestine (Darnaud et al., 2018). The AMPs can attenuate intestinal mucosal barrier injury in diarrheic weaned pigs and infected pigs (Yi et al., 2017). In this study, sow FABO supplementation increased goblet cell numbers and the expression of *pBD-3* and *pBD-2* in the jejunum of offspring. The increased number of goblet cells and expression of AMPs helped to reinforce intestinal mucosal barrier function. Mucosal sIgA blocks pathogen adhesion and neutralizes bacterial toxin, fortifying intestinal immune defense (Russell and Kilian, 2005). Sow FABO supplementation enhanced the level of sIgA in the offspring's jejunum, consistent with Yao et al. (2012), who demonstrated that reducing the maternal dietary n-6 to n-3 PUFA ratio increased circulatory IgG concentration in piglets. Maternally derived colostrum and milk provide essential Ig for the intestinal maturation and disease resistance of piglets (Le Dividich et al., 2005). Sow FABO supplementation improved intestinal barrier integrity and elevated sIgA levels in the intestine of piglets, which might be involved in the high maternal Ig content in colostrum. These findings suggest that maternal FABO supplementation maintains intestinal barrier integrity by improving intestinal immunity.

The regulatory mechanism of intestinal immunity is complex, involving multiple signaling pathways. The IL-22/STAT3 signaling pathway critically regulates innate defense, epithelial regeneration, and the inflammatory response (Ouyang et al., 2011). As an IL-10 cytokine family member, IL-22 binds IL-22R1 to activate the receptor-associated JAK1 and TYK2, triggering STAT3 phosphorylation (Patnaude et al., 2021) and subsequently inducing expression of genes that drive mucus secretion, epithelial proliferation, and wound healing (Arshad et al., 2020). Furthermore, IL-22 upregulation also ameliorates intestinal inflammation and stimulates AMPs synthesis, bolstering epithelial barrier integrity (Sugimoto et al., 2008). Sow FABO supplementation alleviated the decrease of mRNA abundance of *JAK1* caused by LPS challenge, enhanced IL-22 and p-STAT3 protein expression, and p-STAT3:t-STAT3 ratio in the intestine of piglets. In agreement with the findings of this study, Yang et al. (2020) demonstrated that short-chain fatty acid supplementation protected intestines against inflammation by enhancing IL-22 production in mice. Collectively, in this study, the results suggest that maternal FABO supplementation improves offspring intestinal barrier function and reduces the expression of proinflammatory cytokines, which might be involved in regulating IL-22/STAT3 signaling pathway.

## 5 Conclusion

In conclusion, maternal supplementation with 2% FABO increased colostrum concentrations of IgG, IgM, and IgA, and improved the growth performance of offspring. Moreover, dietary supplementation of sows with 2% FABO enhanced intestinal barrier function of piglets, potentially through modulation of the IL-22/STAT3 signaling pathway.

## Credit Author Statement

**Huiling Zhu:** Writing – original draft, Investigation, Data curation. **Yuhui Tang:** Writing – original draft, Investigation. **Kun Qin:** Investigation. **Mengke Yan:** Investigation. **Yu Cao:** Investigation. **Jihua Wang:** Investigation. **Xiao Xu:** Data curation. **Shaokui Chen:** Data curation. **Kan Xiao:** Data curation. **Yulan Liu:** Writing – review & editing, Supervision, Project administration, Funding acquisition.

## Declaration of competing interest

We declare that we have no financial and personal relationships with other people or organizations that can inappropriately influence our work, and there is no professional or other personal interest of any nature or kind in any product, service and/or company that could be construed as influencing the content of this paper. The authors declare the following financial interests/personal relationships which may be considered as potential competing interests: Yu Cao and Jihua Wang are currently employed by Calid Biotech (Wuhan) Co., Ltd., Wuhan, Hubei, China.
