# Supplementary material for: Effects of maternal supplementation with fatty acid-balanced oil on the performance of sows and intestinal barrier function of offspring
Source: Anim Nutr. 2026 Sep 9;27:412–23. doi: 10.1016/j.aninu.2026.04.009 (PMC13677038; doi:10.1016/j.aninu.2026.04.009)
Supplement: Supplementary file 1 [file mmc1.docx]

**Table S1** Abbreviations and full names of genes and proteins.

| Abbreviations | Full names |
| --- | --- |
| **Genes** | |
| *COX-2* | Cytochrome oxidase subunit 2 |
| *HSP70* | Heat shock protein 70 |
| *IL-1β* | Interleukin-1β |
| *IL-6* | Interleukin-6 |
| *IL-10* | Interleulin-10 |
| *IL-22* | Interleukin-22 |
| *IL-22R1* | Interleukin-22 recepror 1 |
| *JAK1* | Janus kinase 1 |
| *pBD-1* | Porcine β defensin 1 |
| *pBD-2* | Porcine β defensin 2 |
| *pBD-3* | Porcine β defensin 3 |
| *PPARγ* | Peroxisome proliferator-activated receptor gamma |
| *STAT3* | Signal transducer and activator of transcription 3 |
| *TLR4* | Toll-like receptor 4 |
| *TNF-α* | Tumor necrosis factor-α |
| *TYK2* | Tyrosinase kinase 2 |
| **Proteins** | |
| p-STAT3 | Phosphor-signal transducer and activator of transcription 3 |
| t-STAT3 | Total-signal transducer and activator of transcription 3 |
| ZO-1 | Zonula occludens-1; |

**Table S2** Primer sequences used for quantitative real-time PCR.

| Genes | Primer sequences (5’-3’) | Accession No. | Product size, bp |
| --- | --- | --- | --- |
| *pBD-1* | F: CGCCTCCTCCTTGTATTC  R: ACACACGCCTTTATTCCTT | NM_213838.1 | 102 |
| *pBD-2* | F: CTTGCCACTGTAACAGGTC  R: GACATAACAGGATGAGGTTGATT | NM_214442.2 | 100 |
| *pBD-3* | F: GCCACTCACAGAACAGCTACC  R: CTGCTTCAGCTTCTGGTTGC | XM_021074698.1 | 163 |
| *IL-1β* | F: GCTAACTACGGTGACAACAATAATG  R: CTTCTCCACTGCCACGATGA | NM_214055.1 | 186 |
| *IL-6* | F: CTCCAAACTGGAGGTGGCG  R: CTGAGCACCCAGTGAATGGT | NM_214403.1 | 114 |
| *IL-10* | F: CCACAAGTCCGACTCAACGA  R: GGCAACCCAGGTAACCCTTA | NM_214041.1 | 267 |
| *HSP70* | F: GCCCTGAATCCGCAGAATA  R: TCCCCACGGTAGGAAACG | NM_001123127.1 | 152 |
| *COX2* | F: ATGATCTACCCGCCTCACAC  R: AAAAGCAGCTCTGGGTCAAA | NM_214321.1 | 284 |
| *IL-22* | F: GCAGGCTTGACAAGTCCAACT  R: GCCTCCTTAGCCAGCATGAA | XM_021091968.1 | 70 |
| *IL-22R1* | F: CGTATCTTCAGCCATCACT  R: GCACTTCTGTAACTCCTCT | XM_021095535.1 | 175 |
| *JAK1* | F: CATTAACAAGCAGGACAACA  R: GACCGTGACAACCATTCT | NM_214114.1 | 179 |
| *TYK2* | F: GCACTGGTGGATGGCTACT  R: TGAGGATGAGGCGGTTGAG | NM_001114670.1 | 205 |
| *STAT3* | F: AGGACATCAGCGGTAAGA  R: AGCGGAGACACAAGGAT | NM_001044580.1 | 133 |
| β-Actin | F: TGCGGGACATCAAGGAGAAG  R: AGTTGAAGGTGGTCTCGTGG | XM_021086047.1 | 216 |

F = forward; R = reverse.
